# The Melanocortin System in Control of Inflammation

**DOI:** 10.1100/tsw.2010.173

**Published:** 2010-09-14

**Authors:** Anna Catania, Caterina Lonati, Andrea Sordi, Andrea Carlin, Patrizia Leonardi, Stefano Gatti

**Affiliations:** ^1^ Center for Preclinical Investigation, Ospedale Maggiore Policlinico, Milano, Italy; ^2^ Department of Internal Medicine, Università di Milano, Italy; ^3^ Center for Surgical Research, Fondazione IRCCS Ca' Granda - Ospedale Maggiore Policlinico, Milano, Italy

**Keywords:** α-, β-, and γ-melanocyte-stimulating hormone (α-, β-, γ-MSH), acute respiratory distress syndrome (ARDS), adhesion molecules, adrenocorticotropic hormone (ACTH), allergic inflammation, brain injury, cyclic 3’, cytokines, endothelial cells, fibroblast, G protein-coupled receptors (GPCRs), gout, inflammatory bowel disease (IBD), keratinocyte, lymphocyte, macrophage, melanocortin receptors, melanocortin receptor accessory protein (MRAP), melanocyte, monocyte, neutrophil, neuroprotection, nuclear factor κB (NF-κB), pro-opiomelanocortin (POMC), reperfusion injury, rheumatoid arthritis, septic shock, signal transducers and activator of transcription (STAT), suppressors of cytokine signaling (SOCS), tumor necrosis factor α (TNF-α)

## Abstract

Melanocortin peptides, the collective term for α-, β-, and γ-melanocyte-stimulating hormone (α-, β-, γ-MSH) and adrenocorticotropic hormone (ACTH), are elements of an ancient modulatory system. Natural melanocortins derive from the common precursor pro-opiomelanocortin (POMC). Five receptor subtypes for melanocortins (MC_1_-MC_5_) are widely distributed in brain regions and in peripheral cells. Melanocortin receptor activation by natural or synthetic ligands exerts marked anti-inflammatory and immunomodulatory effects. The anticytokine action and the inhibitory influences on inflammatory cell migration make melanocortins potential new drugs for treatment of inflammatory disorders. Effectiveness in treatment of acute, chronic, and systemic inflammatory disorders is well documented in preclinical studies. Further, melanocortins are promising compounds in neuroprotection. This review examines the main signaling circuits in anti-inflammatory and immunomodulatory actions of melanocortins, and the potential therapeutic use of these molecules.

**KEYWORDS:** α-, β-, γ-melanocyte stimulating hormone (α-, β-, γ-MSH); acute respiratory distress syndrome (ARDS); adhesion molecules; adrenocorticotropic hormone (ACTH); allergic inflammation; brain injury; cyclic 3',5'-adenosine monophosphate (cAMP); cytokines; endothelial cells; fibroblast; G protein-coupled receptors (GPCRs); gout; inflammatory bowel disease (IBD); keratinocyte; lymphocyte; macrophage; melanocortin receptors; melanocortin receptor accessory protein (MRAP); melanocyte; monocyte; neutrophil; neuroprotection; nuclear factor κB (NF-κB); pro-opiomelanocortin (POMC); reperfusion injury; rheumatoid arthritis; septic shock; signal transducers and activator of transcription (STAT); suppressors of cytokine signaling (SOCS); tumor necrosis factor α (TNF-α)

## MELANOCORTIN PEPTIDES

Melanocortin peptides, the collective term for α-, β-, and γ-melanocyte-stimulating hormone (α-, β-, γ-MSH) and adrenocorticotropic hormone (ACTH), derive from the common precursor pro-opiomelanocortin (POMC)[1]. Melanocortin peptides and their receptors are elements of an ancient modulatory system that appeared very early in evolution. The pituitary of the lamprey, the most ancient of vertebrates, contains recognizable POMC sequences with structural similarity to those of teleosts and higher vertebrates[2].

Post-translational processing of POMC is made by specific enzymes[3]. Prohormone convertase type 1 (PC1, also known as PC3) is a serine-type proteinase that cleaves POMC to produce pro-ACTH and β-lipotropin (β-LPH). PC1 then acts on the pro-ACTH molecule to produce an N-terminal peptide (N-POC) and a joining peptide (JP)[4]. A second serine proteinase called prohormone convertase 2 (PC2) has a number of target substrates and acts on the β-LPH molecule to form γ-lipotropin (γ-LPH) and β-endorphin (β-END)[5]. PC2 also operates on ACTH to produce ACTH 1-17, corticotrophin-like intermediate lobe peptide (CLIP), and α-MSH. Other target sites for PC2 include the cleavage of γ-LPH to form β-MSH and N-POC to form γ-MSH. The predominant site for production of the melanocortin peptides is the pituitary gland, however, extrapituitary sites for melanocortin peptide production are also reported[6].

The natural ACTH is composed of 39 amino acids. α-MSH consists of the first 13 N-terminal amino acid residues of ACTH and is identical in all mammals. It is a basic peptide and is acetylated at the N-terminus and amidated at the C-terminus. β-MSH is a 22-residue peptide. γ-MSH peptides are present in three pharmacologically active forms: $\gamma_1$-MSH, $\gamma_2$-MSH, and $\gamma_3$-MSH. All the melanocortin peptides share a common “core sequence” (His-Phe-Arg-Trp) that is responsible for receptor recognition[3,7].

## MELANOCORTIN RECEPTORS

A major breakthrough in understanding melanocortin action came with the cloning of the melanocortin receptor family[8]. Five subtypes of melanocortin receptors, $MC_1$–$MC_5$, have been identified to date[9,10,11,12,13,14,15]. Melanocortin receptors partly differ in their affinities for natural melanocortins and in tissue distribution. However, melanocortin receptor distinction based on ligand affinity and distribution is largely artificial. With the exception of the $MC_2$ receptor, which is selectively activated by ACTH, the other subtypes recognize all the natural melanocortins. Similarly, although there are differences in tissue distribution, occurrence is redundant and there is large overlap.

The $MC_1$ receptor was the first of the melanocortin receptors to be cloned[8]. $MC_1$ has high affinity for α-MSH and ACTH, and is expressed in a wide range of cells[16], including skin cells (keratinocytes, melanocytes, and fibroblasts), melanoma cells, immune cells (neutrophils, monocytes, dendritic cells, B lymphocytes), glial cells, and endothelial cells. The best recognized function of the $MC_1$ is induction of melanogenesis[17,18]. α-MSH/$MC_1$ interaction in melanocytes promotes intracellular elevation of cyclic 3’,5’-adenosine monophosphate (cAMP), which in turn activates the enzyme tyrosinase, resulting in the synthesis of eumelanin[17,18]. However, $MC_1$ is also expressed in many nonpigmentary cells, and a function for this receptor during inflammation and immunoregulation is now supported by a large number of studies[16].

The $MC_2$ receptor exclusively responds to stimulation by ACTH[7]. This receptor subtype is expressed in the adrenal cortex and adipocytes, and is involved in steroidogenesis[19]. $MC_2$ is the only melanocortin receptor that shows binding selectivity for a specific melanocortin; indeed, the melanotropins α-, β-, and γ-MSH do not activate this receptor and do not promote corticosteroid production.

$MC_3$ is mainly expressed in the central nervous system[20], but also occurs in human B lymphocytes, macrophages, placenta, heart, and gut[21]. It contributes to energy homeostasis[20] and control of inflammation[22].

$MC_4$ is the prevalent melanocortin receptor within the brain[20,23]. It is highly expressed in the hypothalamus, spinal cord, and cortex. This receptor subtype mediates most of the central effects of melanocortins, including neuroprotection[24]. Both the $MC_3$ and $MC_4$ are involved in the regulation of energy homeostasis and feeding behavior[25,26,27].

$MC_5$ is a relatively ubiquitous receptor in peripheral tissues. There is evidence that this receptor subtype participates in control of exocrine secretions[28]. However, data also indicate immunomodulatory functions in B and T lymphocytes, and in mast cell lines[29,30].

Melanocortin receptors belong to the family of seven transmembrane (7TM) G protein-coupled receptors (GPCRs). All natural melanocortin ligands contain a His-Phe-Arg-Trp core sequence that is important for melanocortin receptor recognition[31,32]. The melanocortin receptor system is unique among GPCRs in that there are naturally occurring agonists and antagonists[3]. The melanocortin antagonists, agouti and agouti-related protein (AGRP), are the only two endogenous antagonists of GPCRs identified to date[33,34].

Recent research indicates that, in addition to G proteins, a large number of proteins interact with various GPCRs[35]. These interacting proteins include GPCRs themselves as homo- or heterodimers, multidomain scaffolding proteins, and accessory/chaperone molecules[35]. They provide diverse molecular mechanisms for ligand recognition, signaling specificity, and receptor trafficking. Similar to other GPCRs, melanocortin receptors also utilize accessory/chaperone molecules[36,37,38,39].

Activation of $MC_2$ by ACTH requires an accessory protein called melanocortin 2 receptor accessory protein, or MRAP[37]. MRAP interacts directly with the $MC_2$ and is essential for its trafficking from the endoplasmic reticulum to the cell surface[37]. Individuals lacking MRAP are ACTH-resistant and glucocorticoid-deficient[37]. Human MRAP comprises two splice variants, MRAPα and MRAPβ, and a second gene, MRAP2[38,40,41]. MRAPα and MRAP2 have been found to interact with all five receptor subtypes *in vitro* and, in contrast to the $MC_2$ that requires MRAP for function, these MRAPs reduce $MC_1$, $MC_3$, $MC_4$, and $MC_5$ coupling to adenylyl cyclase[40,42].

Mahogunin ring finger-1 (Mgrn1) is a recently discovered accessory protein for melanocortin signaling[39]. Mahogunin is a RING domain–containing ubiquitin ligase conserved in invertebrate genomes and its absence causes a pleiotropic phenotype known as mahoganoid[39]. Mgrn1 reduces $MC_1$ and $MC_4$ functional coupling to cAMP cascade without decreasing receptor density or intracellular stability. This inhibitory effect occurs upstream of G protein α subunit (Gα) activation of adenylyl cyclase and is abolished when Gα is overexpressed. Therefore, Mgrn1 and Gα appear to compete for binding to melanocortin receptors, suggesting a new mechanism for melanocortin signaling inhibition that involves Gα displacement[43].

## SIGNALING PATHWAYS

Melanocortin receptors are functionally coupled to adenylyl cyclase and mediate their effects primarily by activating a cAMP-dependent signaling pathway. Stimulation of cAMP production by melanocortin receptor ligands causes activation of protein kinase A (PKA) and cAMP response element-binding (CREB) phosphorylation. However, melanocortin signaling is also conveyed through other cAMP-independent pathways[1]. Intracellular free calcium elevation has been detected after $MC_1$ and $MC_3$ stimulation in response to α-MSH[44,45,46]. Further, Janus kinase/signal transducers and activator of transcription (Jak/STAT) activation occurred upon $MC_5$ stimulation[29]. Therefore, evidence suggests the presence of alternative or additional pathways[46]. It is reasonable to believe that activation of these pathways is under physiological control and that individual pathways can communicate with each other, as receptor “cross-talk”. Of interest, if the cAMP pathway is inhibited pharmacologically by the use of agents such as N6-phenylisopropyl adenosine (R-PIA), α-MSH induces an acute intracellular calcium signal[44,47]. These observations suggest that the $MC_1$ receptor is linked to both adenylyl cyclase and the $Ca^{2+}$/diacylglycerol/inositol trisphosphate pathways. The response to R-PIA/α-MSH has been reported in both human melanoma cell types[44] and human keratinocyte cells[47]. Therefore, data on human melanoma cells and keratinocytes suggest that α-MSH acts through a “dominant” cAMP pathway, but under conditions where this pathway is inhibited, a $Ca^{2+}$ signal appears to occur[46].

## MECHANISMS OF THE ANTI-INFLAMMATORY ACTION

The base for the remarkably broad effects of α-MSH on inflammatory mediator production was partly clarified by the discovery that the peptide inhibits activation of the nuclear transcription factor NF-κB[48,49,50,51,52,53,54,55]. The discovery that melanocortins reduce NF-κB activation suggests that any gene under control of NF-κB is under potential regulation by these peptides. NF-κB controls the expression of hundreds of genes that include cytokines, cytokine receptors, chemokines, growth factors, and adhesion molecules[56] . A number of functional studies have shown the link between melanocortins, NF-κB, and the relative expression of several downstream proteins[48,49,50,51,52,53,54,55].

NF-κB is retained in an inactive form in the cytoplasm, bound to members of the IκB inhibitory protein family[56]. Phosphorylation of IκB by various agents such as cytokines, bacterial products, and viruses causes IκB degradation. Subsequently, the free NF-κB is translocated to the nucleus, where it binds to sequences of DNA encoding NF-κB-responsive elements and triggers the transcription of target genes. A signal discovery was that α-MSH down-regulates NF-κB activation induced in monocytes by inflammatory stimuli, including tumor necrosis factor α (TNF-α), endotoxin, ceramide, and okadaic acid[48]. Suppression of NF-κB translocation occurred through generation of cAMP, activation of PKA, and protection of IκBα from phosphorylation[48]. Inhibition of NF-κB activation was subsequently observed in human glioma cells[57], macrophages[55], fibroblasts[58,59], endothelial cells[60], keratinocytes[61], melanocytes[52], olfactory ensheathing cells (OECs)[62], Schwann cells[63], and in human immunodeficiency virus (HIV)–infected monocytes[64]. Experiments on cells transfected with a plasmid vector encoding α-MSH indicate that the peptide can inhibit NF-κB activation in an autocrine fashion[53,54,65].

A further contribution to clarification of the mechanism of action of melanocortins comes from the observation that IL-1 receptor–associated kinase (IRAK)-1, the signal molecule induced by activation of the toll-like receptor 4 (TLR4), is bound to the inhibitory molecule IRAK-M in macrophages treated with α-MSH[66]. Therefore, it appears that inhibition of NF-κB activation occurs at an early step of signal transduction.

Modulatory influences in lymphocytes and dendritic cells suggest that melanocortins could have beneficial effects in immune-mediated inflammation. Research in human lymphocytes indicates that α-MSH exerts an inhibitory action on antigen-stimulated lymphocyte proliferation[67]. Consistent with this observation, α-MSH markedly reduced anti-CD3/CD28–induced proliferation of spleen cells and $CD4^{+}CD25^{-}$ T lymphocytes[68]. Such an inhibitory effect on T-cell proliferation was associated with increased production of interleukin-10 (IL-10) and reduced productions of IL-2 and interferon γ (INF-γ)[68]. In experiments on dendritic cells, α-MSH inhibited cell activity via down-regulation of antigen-presenting and adhesion molecules[69].

With regard to the receptor subtypes responsible for the anti-inflammatory action of melanocortins, the issue is far from being clearly understood. The modulatory signaling pathways are thought to arise predominantly via the $MC_1$ and $MC_3$ receptors in peripheral cells[16,22,70,71,72,73,74] and via activation of $MC_4$ within the brain[75]. In addition, evidence indicates that $MC_5$ receptors participate in modulation of immune-mediated inflammation[29,76]. Significant information on the contribution of individual melanocortin receptors to anti-inflammatory effects is provided by knockout models. When experimental colitis was induced in mice with a frameshift mutation in the $MC_1$ gene, the course of the disease was severely worsened[77]. Vascular inflammation and cell emigration appear to be modulated by the $MC_3$ receptor[21]. Indeed, $MC_3$-null mice showed enhanced plasma extravasation and cell emigration after occlusion and reopening of the superior mesenteric artery[21]. Finally, expression of $MC_5$ appears to be required for protection of the retina from the damage caused by experimental autoimmune uveoretinitis (EAU). Induction of ocular autoantigen-responsive $CD4^{+}$ regulatory T cells in the post EAU spleen likewise needs a functional $MC_5$[78].

## THERAPEUTIC POTENTIAL IN INFLAMMATORY DISORDERS

The anticytokine action and the inhibitory effect on inflammatory cell migration make melanocortins potential new drugs for treatment of inflammatory disorders[16,79,80,81] (Fig. 1).

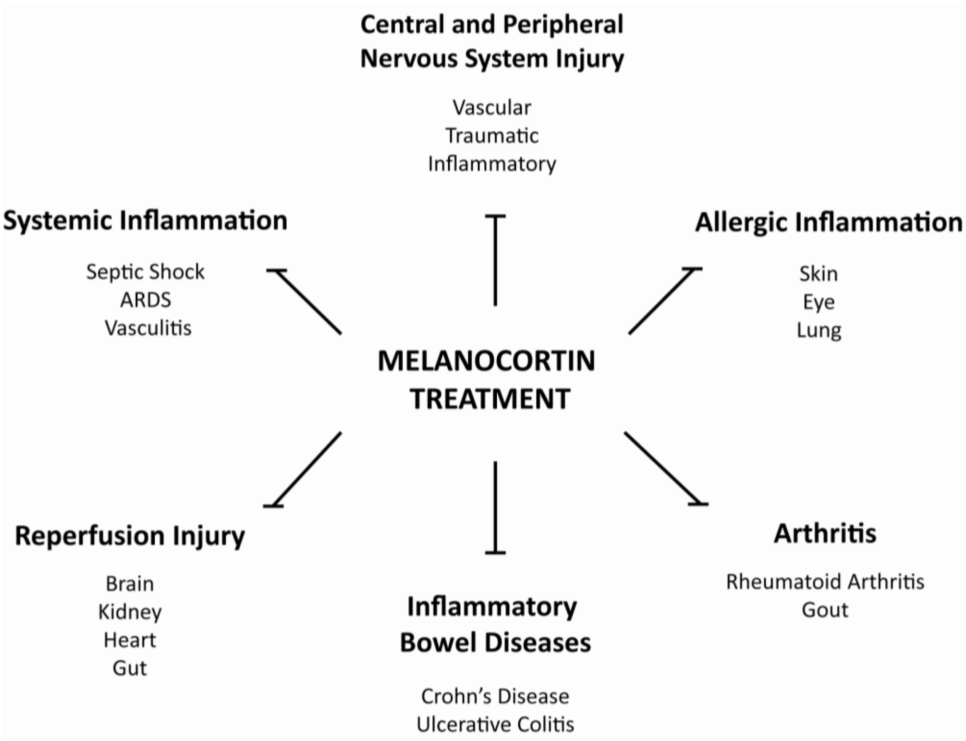

**FIGURE 1.** Beneficial effects of melanocortin treatment are well documented in preclinical models of inflammatory disorders.

### Inflammation in Central and Peripheral Nervous System

Melanocortins reduce production of cytokines and other inflammatory mediators in models of neural damage *in vitro* and *in vivo*[24]. As natural and synthetic melanocortins have neuroprotective action in many preclinical models of neural injury of vascular, inflammatory, and traumatic origin, these compounds are promising candidates for treatment of central and peripheral nervous system injury[24].

Regardless of the underlying etiology, damage to oligodendrocytes and Schwann cells is primarily caused by proinflammatory cytokines and free radicals produced by activated microglia, macrophages, and astrocytes[82]. In experiments *in vitro*, α-MSH (1-13), α-MSH (11-13), and ACTH (1-24) inhibited production of TNF-α, IL-6, and nitric oxide (NO) in a cultured murine microglial cell line stimulated with lipopolysaccharide (LPS) plus IFN-γ[83] or β-amyloid plus IFN-γ[84]. Further, when stimulated with LPS plus IFN-γ, microglia increased release of α-MSH and immunoneutralization of the peptide enhanced production of inflammatory mediators[83]. In astrocytes stimulated with LPS and IFN- γ, α-MSH inhibited expression of inducible nitric oxide synthase (iNOS) and cyclooxygenase 2 (COX-2), and reduced production of the inflammatory mediators NO and prostaglandin $E_2$[85]. Further, treatment with α-MSH prevented LPS/IFN-γ–induced apoptosis of astrocytes; this effect was mediated by modulation of proteins of the Bcl-2 family[85].

Axon regeneration in the central nervous system is enhanced by the presence of OECs that have the capacity to support regeneration and myelination of long tract axons[86]. These cells are, therefore, candidates for cell-based neuroprotective therapies, but proinflammatory cytokines produced during neural injury could damage them. In research on primary rat OECs, α-MSH inhibited TNF-α– and IFN-γ–induced activation of NF-κB[62]. It appears, therefore, that outcome of OEC-based therapy could be improved by concomitant treatment with a melanocortin.

Protective effects of α-MSH were also observed in glial cells of the peripheral nervous system. Indeed, the peptide inhibited NF-κB activation in cultured rat primary Schwann cells stimulated with TNF-α and IFN-γ[63]. This effect could be very important to improve the healing responses and promote nerve regeneration after peripheral nerve injury.

## Systemic Inflammation

Disorders such as sepsis syndrome, septic shock, acute respiratory distress syndrome (ARDS), autoimmune vasculitis, and several other conditions are collectively classified as "systemic inflammation". The systemic inflammatory response is initially useful to limit infection, but can also be detrimental and cause organ failure. Modulation of the systemic reactions is, therefore, important in order to restrict mediator release and cell activation that could cause harmful consequences. Preclinical evidence indicates that melanocortins are effective modulators of these reactions[87].

Seminal experiments demonstrated that α-MSH reduces the acute phase response to endotoxin[88]. Subsequently, it became clear that α-MSH given centrally or peripherally modulates many aspects of response to endotoxemia[89] and improves survival in models of septic shock[90,91].

ARDS is a severe complication of pneumonia, sepsis, trauma, and inhaled irritants. It is characterized by production of proinflammatory mediators, massive neutrophil influx into the lung, and damage to lung epithelium. Research on lung injury induced by endotracheal instillation of endotoxin in rats showed that treatment with α-MSH greatly reduces leukocyte concentration in the bronchoalveolar lavage fluid[91]. These salutary effects of α-MSH were confirmed and extended in lung injury caused by intratracheal instillation of bleomycin[92]. Treatment with α-MSH modulated expression of genes involved in stress response, fluid homeostasis, and inflammation[92].

The Shwartzman reaction is a potentially lethal generalized thrombohemorrhagic hypersensitivity response that occurs after sequential injections of LPS[93]. Persistent expression of vascular adhesion molecules contributes to enhanced diapedesis and activation of leukocytes, which subsequently leads to hemorrhagic vascular damage. In LPS-induced cutaneous vasculitis in mice, a single injection of α-MSH significantly suppressed the deleterious vascular damage and hemorrhage by inhibiting the sustained expression of vascular E-selectin and vascular cell adhesion molecule (VCAM)-1[94]. These observations indicate that α-MSH may have therapeutic potential for the treatment of vasculitis associated with Gram-negative infections.

## Reperfusion Injury

Tissue damage that occurs during blood reperfusion after ischemia (RI) is a serious problem in many vascular disorders and reperfusion procedures. Treatment with α-MSH and other melanocortins reduced ischemia and reperfusion injury in heart[95,96,97], brain[98,99,100], kidney[101,102,103,104,105,106,107,108], and gut[51]. α-MSH administration prevented most of the changes induced by genetic mismatch in allografts and improved antigen-independent gene expression linked to mechanical damage and reperfusion in syngenic transplantation[109,110]. The broad anti-inflammatory effects of melanocortins, including those in RI, mostly depend on the capacity of the peptides to inhibit IκBα phosphorylation and, consequently, NF-κB translocation to the nucleus[48,51,52,53]. In addition, activation of the cholinergic anti-inflammatory pathway appears to contribute significantly to the protective effects of melanocortins during RI[111]. Functional $MC_3$ receptors appear to be required for inhibition of vascular inflammation during RI[21].

Recent research found that α-MSH induces phenotype changes in the heart that resemble ischemic preconditioning[112]. Ischemic preconditioning is a potent cardioprotective mechanism induced by repetitive sublethal ischemic events[113]. Treatment of rats with α-MSH was associated with early and marked increase in IL-6 mRNA in the heart[112]. This was followed by STAT3 phosphorylation and induction of suppressor of cytokine signaling 3 (SOCS3). These observations indicate that α-MSH induces phenotype changes that closely resemble ischemic preconditioning and likely contribute to its established protection against reperfusion injury. Because SOCS proteins are cytokine antagonists that act as a negative feedback that inhibits cytokine signal transduction[114], induction of SOCS3 could be a relevant component in the anti-inflammatory action of α-MSH.

## Inflammatory Bowel Diseases

The etiology of inflammatory bowel diseases (IBD) is believed to depend on a combination of genetic and environmental factors. Because of the critical role of enhanced NF-κB activation for development of IBD, this transcription factor is the main target in present IBD treatments[115,116] and in development of novel therapeutic approaches[117,118,119]. Research on experimental IBD showed that treatment with α-MSH effectively reduces severity of experimental colitis[120,121]. α-MSH was administered to mice with dextran sulfate–induced colitis, a model of IBDs[121]. The treatment had marked salutary effects: it reduced the appearance of fecal blood, inhibited weight loss, and prevented disintegration of the general condition of the animals. Consistent with these observations, α-MSH administration reduced the colonic macroscopic lesions in both acute and chronic colitis induced by trinitrobenzene sulfonic acid[120]. α-MSH also had beneficial effects on endotoxin-induced intestinal lesions[122]. α-MSH treatment reduced severity of the lesions macroscopically and microscopically, although the protective effect was mainly confined to the distal ileum. The salutary effect probably involved COX-1–derived prostaglandins in that it was reversed by pretreatment with the nonselective COX-1 inhibitor indomethacin[122]. Of interest, it appears that the endogenous melanocortin system protects the host from IBD development. Indeed, research on colitis in mice with a frameshift mutation in the MC1 gene showed significant worsening in disease severity[77].

## Arthritis

In 1949, long before melanocortin receptors were discovered, Hench and colleagues reported the therapeutic efficacy of ACTH in rheumatoid arthritis patients[123]. Soon after, in 1950, ACTH effectiveness was reported in patients suffering from gouty arthritis[124]. At that time, the therapeutic action of ACTH was thought to depend on cortisol induction. A more recent clinical study in gouty arthritis patients confirmed the efficacy of ACTH[125]; this controlled study suggested the existence of other molecular mechanisms in addition to adrenal gland activation and increase in circulating cortisol. ACTH is currently used in treatment of gouty arthritis in patients manifesting intolerance to nonsteroidal anti-inflammatory drugs[126].

The therapeutic potential of melanocortins in different forms of inflammatory arthritis is well documented in experimental models[127,128,129,130,131]. Local injections of ACTH in rats with monosodium urate crystal–induced arthritis had anti-inflammatory effects. ACTH treatment was also effective in adrenalectomized rats, but was prevented by concomitant administration of the $MC_3$ antagonist SHU9119[129]. The $MC_3$ selective agonist γ2-melanocyte-stimulating hormone likewise reduced arthritis in this gout model[129]. Treatment with α-MSH significantly reduced joint pathology in rats with adjuvant-induced arthritis, which is a preclinical model of rheumatoid arthritis[128]. Effectiveness of α-MSH on joint inflammation was similar to that of prednisolone. However, whereas there was a significant weight reduction in prednisolone-treated and -untreated animals, α-MSH–treated animals maintained their weight over the observation period[128].

Recent research indicates that human articular chondrocytes are target cells for α-MSH[132]. $MC_1$ expression was detected in articular chondrocytes *in vitro* and in articular cartilage *in situ*. In addition, expression of transcripts for $MC_2$, $MC_5$, POMC, and PCs was detected in articular chondrocytes. Stimulation with α-MSH increased the levels of intracellular cAMP in chondrocytes. Both messenger RNA and protein expression of various proinflammatory cytokines, collagens, matrix metalloproteinases (MMPs), and SOX9 were modulated by α-MSH[132].

## Allergic Inflammation

There is evidence that melanocortins inhibit acute inflammation in the skin. Early studies on anti-inflammatory influences of α-MSH indicated that the peptide inhibits increase in capillary permeability induced by intradermal injections of histamine or IL-1 in rabbits[133]. Subsequently, modulatory effects of α-MSH peptides were confirmed in acute skin inflammation induced by nonspecific irritants and cytokines[134,135,136,137].

α-MSH inhibits cutaneous immune-mediated responses *in vivo*[134,138,139,140,141]. When applied epicutaneously, α-MSH inhibited both induction and elicitation of contact hypersensitivity responses in mice[141]. Systemically administered α-MSH likewise promoted induction of hapten-specific tolerance[140]. Regional lymph node cells obtained from α-MSH–treated mice after resensitization were unable to produce IL-2 in response to trinitrobenzosulfonic acid. *In vivo* tolerance induction by α-MSH could be abrogated by the administration of an anti–IL-10 antibody at the site of sensitization[140]. Therefore, contact hypersensitivity and acute inflammatory reactions in the skin could be a potential therapeutic target for α-MSH peptides.

In addition to the skin, α-MSH had marked modulatory action in allergic lung inflammation[142,143]. In experiments on allergic bronchial asthma, α-MSH suppressed allergen-specific IgE, $IgG_1$, and $IgG_{2a}$ Ab production, and caused a marked suppression in airway eosinophilia. These effects were mediated by enhanced IL-10 production[143]. In models of allergic and nonallergic lung inflammation, melanocortins inhibited leukocyte accumulation[142]. Such protective effects were associated with activation of the $MC_3$[142].

Beneficial effects of α-MSH have been reported in autoimmune eye disorders[30,78,144]. In autoimmune uveoretinitis, α-MSH converted a population of effector T cells polarized to mediate hypersensitivity into a population of immunoregulatory T cells. Such effector T cells showed similar phenotype relative to inactivated T cells[30,145]. Protective effects of α-MSH in autoimmune uveoretinitis require a functional $MC_5$ receptor[78].

## CONCLUSIONS

The presence of melanocortins in primitive animals was paradoxically detrimental to research in higher organisms. It has not been long since melanocortins were considered an "evolutionary remnant" and the true potential of this system overlooked. Although adrenal, pigmentary, and behavioral effects of melanocortins have been known for over 50 years, the discovery that melanocortins have potent modulatory effects on host responses is much more recent. The anticytokine action and the inhibitory effect on inflammatory cell migration make melanocortins potential new drugs for treatment of acute, chronic, and systemic inflammation. The neuroprotective action in vascular and inflammatory brain injury is likewise well established. Melanocortins could form a novel class of therapeutic agents for several human disorders.

## ACKNOWLEDGMENTS

Research in Dr. Catania's laboratory is supported by funds of "Fondazione IRCCS Ca' Granda - Ospedale Maggiore Policlinico" and "Progetto Fondazione Fiera Milano".
